# Precision Nutrition and Metabolic Syndrome Management

**DOI:** 10.3390/nu11102411

**Published:** 2019-10-09

**Authors:** Pedro González-Muniesa, J. Alfredo Martínez

**Affiliations:** 1 Centre for Nutrition Research, School of Pharmacy and Nutrition, University of Navarra, 31008 Pamplona, Spain; pgonmun@unav.es; 2 Department of Nutrition, Food Science and Physiology, School of Pharmacy and Nutrition, University of Navarra, 31008 Pamplona, Spain; 3 IDISNA, Navarra’s Health Research Institute, 31008 Pamplona, Spain; 4 CIBERobn Physiopathology of Obesity and Nutrition, Centre of Biomedical Research Network, ISCIII, 28029 Madrid, Spain; 5 Precision Nutrition Program, IMDEA Food, 28049 Madrid, Spain

**Keywords:** metabolism, insulin resistance, pathology, therapy, human

The journal *NUTRIENTS* published some time ago a special issue about “Precision Nutrition and Metabolic Syndrome Management”, which included a series of articles about the role of bioactive compounds, amino acids/proteins and fatty acids for personalized nutritional applications [1]. In such volume, the emerging concept of precision nutrition was developed in two specific reviews. These articles explain global personalized nutrition in the management of metabolic syndrome features such as obesity, diabetes, dyslipemia, hypertension and other cardiovascular complications [2], and individualized lipid metabolism targeting in cancer [3]. In particular, they describe the role of nutrigenetics, deep phenotyping, family and personal clinical cues, perinatal nutrition and a wide spectrum of data concerning metabolic personalization through omics technologies (metabolomics, epigenomics, metagenomics, and others), whose possibilities were appraised. Indeed, the conceptual framework understanding precision nutrition is based on assessing the interindividual heterogeneity caused by genetic/epigenetic dissimilarities, the lifestyle and environmental exposome diversity, microbiome variations, and singular behavioral/psychological features [4] with clear implications for public health to harmonize personal and population challenges and applications [5].

In that context, precision nutrition and medicine should consider updated individualized nutritional status indicators for metabolic care and clinical nutrition [6], not only in patients suffering chronic diseases [7] or systemic inflammatory disturbances [8], but also in healthy subjects [9]. Similarly, it is necessary to take into account the development of new transcriptomic biomarkers [10], lipidomic and metabolomic tools [11,12], pharmacogenetic approaches [13] and metagenomic applications [14]. Also, the origins and developmental issues concerning health and disease or epigenetics in line with the developmental origins of health and disease (DOHaD) theory [15] are of interest in precision nutrition [16,17], including specific aspects on obesity [18] and metabolic syndrome features related to liver disease [19].

In the last two years, precision nutrition investigations considering an individual’s facets such as age and gender [20], and nutrigenetic interactions associated to genetic variants involving micronutrients such as vitamin E [21], minerals such as iron [22] or caffeine consumption [23] have been addressed by the Journal NUTRIENTS. Furthermore, concerning clinical precision perspectives also the pages of the journal have published articles related with the role of the microbioma on precision nutrition [24], the development of personalized biomarkers [25], targeted metabolomics [26], applications for weight management [27], on food allergies [28], or a role on inflammation [29].

Indeed, individualized nutrition integrates information based on genetic/epigenetic background. Nevertheless, personalized clinical and phenotypical features including own psychological/personality patterns, specific food allergies and intolerances, differential cultural, social and environmental backgrounds, drug-side effects, personal dietary preferences as well as singular lifestyle and environmental factors need to be addressed for a solid precision nutrition and medicine. Ethical and regulatory issues are needed to be developed and implemented for a smooth growing of nutrition endeavors and methodologies, where important achievements and challenges are expected for combining public and personal health benefits.
